# A GPT‐4o‐Powered Framework for Identifying Cognitive Impairment Stages in Electronic Health Records

**DOI:** 10.1002/alz70857_107622

**Published:** 2025-12-25

**Authors:** Yu Leng, Yignan He, Samad Amini, Colin G. Magdamo, Ioannis Paschalidis, Shibani Mukerji, Lidia Maria V. Moura, M. Brandon Westover, Ana‐Maria Vranceanu, Christine S Ritchie, Deborah Blacker, John R Dickson, Sudeshna Das

**Affiliations:** ^1^ Massachusetts General Hospital, Boston, MA, USA; ^2^ Massachusetts General Hospial, Boston, MA, USA; ^3^ Boston University, Boston, MA, USA; ^4^ Beth Israel Deaconess Hospital, Boston, MA, USA; ^5^ Massachusetts General Hospital (MGH), Boston, MA, USA

## Abstract

**Background:**

Alzheimer's Disease and Related Dementias (ADRD) present a significant public health challenge, emphasizing the need for timely and accurate cognitive impairment (CI) diagnosis. While electronic health records (EHRs) contain valuable cognitive health data, much of this information is embedded in unstructured clinical notes. Advances in natural language processing (NLP) and large language models (LLMs) offer promising solutions, yet the application of models like GPT‐4o for CI identification and staging in EHRs remains underexplored.

**Method:**

We developed a GPT‐4o‐powered framework for CI staging, integrating data querying, feature extraction, and classification. The framework was evaluated on 1002 Medicare patients from Mass General Brigham (MGB), with expert‐adjudicated labels for Cognitively Unimpaired (CU), Mild Cognitive Impairment (MCI), or Dementia. To extract clinically relevant information, the framework employed GPT‐4o to generate multi‐note summaries, compared against keyword‐based sentence extraction. GPT‐4o was further employed for ordinal CI classification, producing a “summary of summaries” along with a confidence level for its final decision. Performance was benchmarked against three alternative models using USE and DementiaBERT embeddings. The framework also integrated structured answer templates, retrieval‐augmented generation (RAG), and CDR domain counts with confidence levels for automated Clinical Dementia Rating (CDR) scoring, using 769 visit notes from the Massachusetts General Hospital (MGH) memory clinic. Evaluation metrics included weighted Cohen's kappa, Spearman's Rank Correlation, and Baccianella's MSE.

**Result:**

The framework demonstrated high accuracy in CI staging (weighted Cohen's kappa = 0.95, Spearman correlation = 0.93, Baccianella's MSE = 0.02), outperforming traditional feature extraction and embedding‐based models. A confidence level stratification analysis showed that GPT‐4o excelled in cases it rated with high confidence. For CDR scoring, domain counts with confidence levels yielded the best results (weighted Cohen's kappa = 0.83). CDR domain documentation in the notes significantly predicted GPT‐4o's confidence in assigning global CDR.

**Conclusion:**

Our GPT‐4o‐powered framework achieves high performance in CI classification, outperforming traditional embedding models and demonstrating potential for automated chart review. However, clinical deployment requires careful consideration of misclassification risks, making a human‐in‐the‐loop approach essential for reliability and safety. This hybrid model underscores AI's role in dementia diagnosis while ensuring interpretability and risk mitigation in real‐world applications.

